# A systematic review of associations between functional MRI activity and polygenic risk for schizophrenia and bipolar disorder

**DOI:** 10.1007/s11682-018-9879-z

**Published:** 2018-05-10

**Authors:** Zalina Dezhina, Siri Ranlund, Marinos Kyriakopoulos, Steve C. R. Williams, Danai Dima

**Affiliations:** 1 Department of Neuroimaging, Institute of Psychiatry, Psychology and Neuroscience, King’s College London, London, UK; 2 National and Specialist Acorn Lodge Inpatient Children Unit, South London and Maudsley NHS Foundation Trust, London, UK; 3 Department of Child and Adolescent Psychiatry, Institute of Psychiatry, Psychology and Neuroscience, King’s College London, London, UK; 4 Department of Psychology, School of Arts and Social Sciences, City, University of London, 10 Northampton Square, London, EC1V 0HB UK

**Keywords:** Polygenic risk score (PGRS), Functional magnetic resonance imaging (fMRI), Schizophrenia, Bipolar disorder, Psychosis, Cognition

## Abstract

Genetic factors account for up to 80% of the liability for schizophrenia (SCZ) and bipolar disorder (BD). Genome-wide association studies have successfully identified several genes associated with increased risk for both disorders. This has allowed researchers to model the aggregate effect of genes associated with disease status and create a polygenic risk score (PGRS) for each individual. The interest in imaging genetics using PGRS has grown in recent years, with several studies now published. We have conducted a systematic review to examine the effects of PGRS of SCZ, BD and cross psychiatric disorders on brain function and connectivity using fMRI data. Results indicate that the effect of genetic load for SCZ and BD on brain function affects task-related recruitment, with frontal areas having a more prominent role, independent of task. Additionally, the results suggest that the polygenic architecture of psychotic disorders is not regionally confined but impacts on the task-dependent recruitment of multiple brain regions. Future imaging genetics studies with large samples, especially population studies, would be uniquely informative in mapping the spatial distribution of the genetic risk to psychiatric disorders on brain processes during various cognitive tasks and may lead to the discovery of biological pathways that could be crucial in mediating the link between genetic factors and alterations in brain networks.

## Introduction

Psychosis is a serious mental illness characterized by delusions, hallucinations and disorganized thinking or behavior. Schizophrenia (SCZ) and bipolar disorder (BD) are the two most common psychiatric disorders associated with psychotic symptoms. Like most complex disorders of non-Mendelian inheritance they are the product of a combination of genetic and environmental factors. Heritability has been calculated as high as 80% for SCZ (Cardno et al. 1999) and 93% for BD (Kieseppa et al. 2004). Both are polygenetic illnesses with thousands of genetic risk factors interacting, each with low to moderate effect (Geschwind and Flint 2015; Kerner 2015; Lichtenstein et al. 2009). Results from genome wide association studies (GWAS) support the idea that thousands of common variants with very small effect sizes are part of the genetic architecture of these disorders (Ripke et al. 2014; Sklar et al. 2011). However, to achieve sufficient statistical power these studies require very large sample sizes, and therefore make replication difficult (Geschwind and Flint 2015). This problem has served as a motivation for the creation of the Psychiatric Genomics Consortium (PGC), which combines genomic data for psychiatric disorders across many studies to perform large scale GWAS analyses and promote rapid progress in this area (Sullivan 2010; Sullivan et al. 2017). The PGC is now a powerful international collaboration working to identify common genetic variations that contribute to psychiatric illnesses, including BD and SCZ. The largest SCZ GWAS to date includes 36,989 cases and 113,075 controls and has identified 125 genetic loci for SCZ (Ripke et al. 2014). Recently, findings from BD GWAS revealed 30 significant single nucleotide polymorphisms (SNPs), including previously reported risk loci such as the CACNA1C gene (Mühleisen et al. 2014; Sklar et al. 2011; Stahl et al. 2017).

**Fig. 1:**
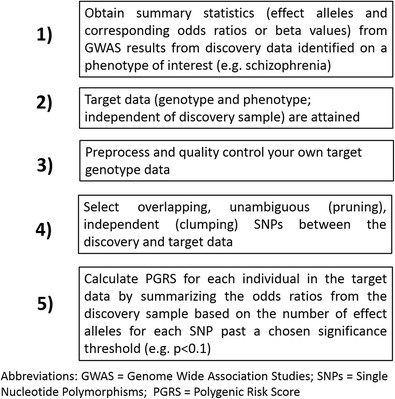
Polygenic risk scoring pipeline

The understanding that each risk SNP may have a very small additive effect across the whole genome became a major driving force to shift the attention from candidate gene (Erk et al. 2013; Paulus et al. 2014; Rasetti and Weinberger 2011) to polygenic models of SCZ and BD (Rucker and McGuffin 2010; Dima and Breen 2015). The polygenic risk score (PGRS) was developed by the Psychiatric Genomics Consortium to measure aggregate genetic risk, based on GWAS results (Purcell et al. 2009). Instead of exploring each genetic risk variant individually, the idea is to link all genetic markers and see how a cumulative effect gives evidence of association with a disorder. First, effect sizes are estimated for each single nucleotide polymorphism (SNP) that is associated with a trait in a large training sample. Then, in an independent replication sample, the PGRS is constructed for each individual and tested for association with the variable or trait of interest (Fig. 1). The use of PGRS increases the power and predictive accuracy of genetic analyses compared to candidate gene studies and sheds new light on the understanding of complex traits (Dudbridge 2013). Dudbridge showed that adequate levels of prediction of complex traits may only be approached when PGRS are estimated from very large samples and that this prediction will become more feasible as sample sizes continue to grow.

A promising area for the application of the PGRS method is the emerging field of research on imaging genetics, which provides opportunities to investigate neurophysiological and neuroimaging markers of genetic risk for psychotic disorders (Bigos and Weinberger 2010; Pezawas and Meyer-Lindenberg 2010). The main aim of imaging genetics is to detect genetic factors that may influence the structure and function of the brain, and thus improve understanding of how this interaction affects cognitive and emotional processes (Hashimoto et al. 2015; Bogdan et al. 2017; Mufford et al. 2017). There has also been success in finding genetic associations for sub-cortical brain volumes through ENIGMA (Stein et al. 2012; Hibar et al. 2015, 2017) and subcortical and cortical volumes through the UK Biobank (Elliott et al. 2017). Until recently, the imaging genetics field for SCZ and BD disorders was dominated by the candidate gene approach, with the underlying problem that candidate genes explain only a very small fraction of the risk for the disorders (Farrell et al. 2015), however interest in PGRS is growing rapidly. Neuroimaging PGRS results are often inconsistent and there is a need for a review on this topic to synthesize findings to date. We aimed to systematically review studies applying PGRS for SCZ and BD to functional magnetic resonance imaging (fMRI) data to investigate the effects of genetic risk on brain function.

## Methods

Recommendations from the Preferred Reporting Items for Systematic Reviews and Meta-analyses (PRISMA) guidelines were used to perform the search strategy (Moher et al. 2015).

### Selection criteria and study selection

The inclusion criteria were: (i) studies including fMRI data, (ii) PGRS calculated from the SCZ and BD summary statistics produced by the PGC.

**Fig. 2:**
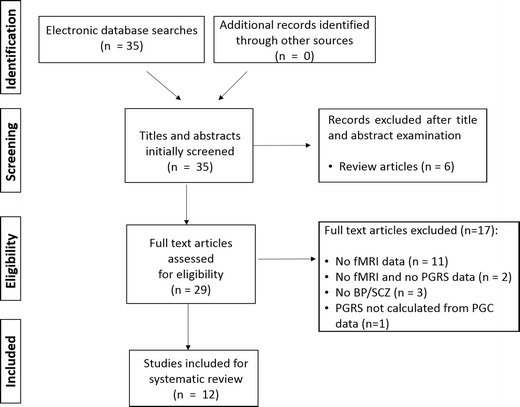
Study selection process

All studies that did not meet inclusion criteria were excluded (Fig. 2). After inspection for duplicates, the titles and abstracts of all studies were reviewed independently by three authors (ZD, SR, DD). The final decision for inclusion or exclusion of the publications was made based on a review of the full texts.

Initially, our goal was to conduct a meta-analysis to integrate data from the different studies. However, we could not proceed with this because there was an insufficient number of studies using similar fMRI paradigms for each PGRS (SCZ and BP) and studies used different levels of fMRI analysis (whole-brain vs region-of-interest).

### Search strategy

To identify studies, we searched PubMed, Scopus, Web of Science and Medline using the following terms: (Polygenic Risk Score OR Cumulative Genetic Risk OR Polygenic Profile) AND (neuroimaging OR fmri OR imaging) AND (Psychosis OR Schizophrenia OR Psychotic disorders OR Bipolar). Moreover, the references of all articles identified were carefully searched for additional studies. We reviewed all papers published in the English language up to October 3rd, 2017.

### Recorded variables, data extraction and analysis

The following variables were obtained from all articles included in the systematic review: Name of the author, number of participants, fMRI task, type of analysis (whole-brain or region-of-interest), main findings including association (positive vs negative), laterality, brain area and MNI coordinates.

After careful inspection of all relevant information, studies were grouped by PGRS for SCZ or BD and classified according to fMRI task. Additionally, three studies were identified that used a PGRS score for psychosis or cross psychiatric disorder that incorporated SCZ and BD summary genetics statistics.

## Results

### Identified studies

The search of the literature identified 35 records. After title and abstract examination, 6 review articles were excluded. Next, 29 potentially relevant studies were carefully evaluated. Seventeen studies that did not contain fMRI data and/or PGRS calculated for SCZ or BD were excluded. One study (Walton et al. 2013) did calculate PGRS SCZ not using PGC summary data but was excluded from the systematic review because the same participants with the same fMRI task were tested with PGRS SCZ scores from the PGC in another study that we have included in our review (Walton et al. 2014). These two studies showed similar results. Thus, 12 papers were included in the systematic review (Fig. 1).

Studies were classified according to PGRS calculated for SCZ, BD or psychosis and cross psychiatric disorders. The studies included in the systematic review describe seven studies with PGRS for SCZ (Table 1), four studies with PGRS for BD (Table 2), one study with PGRS for psychosis and two studies with PGRS using Cross Disorder summary statistics (Table 3). Eleven studies used task fMRI (Cosgrove et al. 2017; Dima et al. 2016; Erk et al. 2017; Kauppi et al. 2015; Lancaster et al. 2016a, b; Rampino et al. 2017; Tesli et al. 2015; Walton et al. 2014; Whalley et al. 2012, 2015), and one used resting state fMRI data (Wang et al. 2017).

**Table 1:** Studies including the polygenic risk score (PGRS) for schizophrenia (SCZ)

Reference	Sample	fMRI Task	Contrast	Analysis	Association with PGRS / PGRS Threshold	Laterality	Brain Region	MNI coordinates
(Cosgrove et al. 2017)	86 HC 70 HC Irish-born paternal and maternal grandparents Mean age: 35.20 (SD = 12.04) Females: 51.6%	Spatial Working Memory	3 dots >1 dot	Whole-brain *P* < 0.05 FWE, cluster level	+ / *P* = 10^−5^	R	Inferior Occipital Gyrus	48, −76, −2
							Middle Temporal Gyrus	3, −34, 16
		Face Processing Task	3 contrast (angry faces, neutral faces, baseline)	Whole-brain P < 0.05 FWE, cluster level	NS / P = 10^−5^	NA	NA	NA
(Erk et al. 2017)	271–296 HC without psychosis family history and 195–214 HC with psychosis family history German volunteers, European origin Mean age: 32.2. (SD = 10.2) Females: 58%	Episodic Memory (recognition)	Memory > control	ROI P_FWE (ROI)_ < 0.05	– / P = 0.05	L	Pregenual Anterior Cingulate Cortex	−3, 26, −11
		Theory of Mind	Mentalizing > control	ROI P_FWE (ROI)_ < 0.05	+ / P = 0.05	L	Posterior Cingulate Cortex	−9, −55, 22
		Working Memory (N-back task)	2 back >0 back	ROI P_FWE (ROI)_ < 0.05	NS / P = 0.05	NA	NA	NA
		Reward Processing (Monetary Incentive Delay Task)	Anticipation of monetary win/loss > anticipation of neutral outcomes	ROI P_FWE (ROI)_ < 0.05	NS / P = 0.05	NA	NA	NA
		Face Matching Task	Angry/fearful faces > shapes	ROI P_FWE (ROI)_ < 0.05	NS/ P = 0.05	NA	NA	NA
(Lancaster et al. 2016a)	83 HC Caucasian Mean age: 23.95 Females: 53%	Probabilistic Decision Making	Shift > stay	Whole-brain P_FWE-WHOLEBRAIN_ corrected	– / P = 0.5	R	Frontal Pole	34, 58, 0
				ROI P_FWE-ROI_ corrected	– / P = 0.5	L	Ventral Striatum	−4, 6, −12
(Kauppi et al. 2015)	63 SCZ and 118 HC Norwegian Data Mean age: Patients: 32.9 (SD = 7.9) Controls: 34.9 (SD = 8.5) Females Patients: 31.7% Controls: 39.8%	Working Memory (N-back task)	2 back >0 back	Whole-brain cluster-level correction z-value >2.3 and a corrected cluster significance threshold of *P* = .05	– / *P* = 0.05	R	Inferior Frontal Gyrus	48, 18, −10
							Medial/Superior Prefrontal Cortex	38, 46, −16
							Middle Temporal Gyrus	72, −40, −4
			2-back > baseline	Whole-brain cluster-level correction z-value >2.3 and a corrected cluster significance threshold of P = .05	– / P = 0.05	R	Middle Temporal gyrus	66, −44, −6
						R	Anterior Cingulate Gyrus	4, 22, 32
						R	Inferior Frontal Gyrus	50, 14, −8
						R	Frontal Pole	26, 34, 22
						R	Middle Temporal Gyrus	58, −22, −12
						L	Post Cingulate	−6, −24, 30
						R	Middle Lateral Ventricle	4, 22, 6
						R	Inferior Frontal Gyrus	46, 30, 10
						L	Putamen	−22, 6, −10
(Rampino et al. 2017)	Discovery 151 HC Replication 51HC Caucasian Mean Age: Discovery: 26 (SD = 10.7) Replication: 25 (SD = 6.1) Females: Discovery: 52% Replication: 49%	Variable Attention Control (VAC task)	3 load levels conditions (high, intermediate, low)	Whole-brain P < .05, small volume FWE corrected	PGRS-SCZ + / P = 10^−8^ NS PGRS-SCZ_Glu_ + / *P* = 10^−8^	L	Superior Frontal Gyrus	−22, 48, 48Discovery −30, 50, 40 Replication
(Walton et al. 2014)	92 SCZ and 114 HC Caucasian descent Mean Age: Patients: 34.23 Controls: 32.49 Females: Patients: 25% Controls: 40.4%	Working Memory (Sternberg Item Recognition Paradigm)	Average load 1, 2, and 3	Whole-brain z value of 2.3 and Bonferroni corrected with a P value of .007 (.05/7)	+ / P = 0.01	L	Dorsolateral/Ventrolateral Prefrontal Cortex	−6, 38, 48
							Dorsolateral Prefrontal Cortex	−12, 48, 30
							Anterior Cingulate Cortex	−4, 46, −18
(Wang et al. 2017)	Dataset 1: 360 HC Dataset 2: 323 HC Chinese Mean Age: Dataset 1: 19.4 (SD = 1.1) Dataset 2: 22.7 (SD = 2.5) Females: Dataset 1: 48.3% Dataset 2: 51.4%	Resting state fMRI (functional connectivity)	NA	ROI voxel-level of P < 0.05 and cluster size >90 voxels to reach a cluster level significance of alpha <0.05	– / *P* = 0.05	L	Dorsolateral Prefrontal Cortex with Insula	−42, 46, 13
					+ / P = 0.05	L	Angular Gyrus with Insula	−54, −62, 28

*HC* Healthy Controls, *L* Left hemisphere, *NA* Not Applicable, *NS* Not Significant, *R* Right hemisphere, *ROI* Region of Interest, *SCZ* Schizophrenia

**Table 2:** Studies including the polygenic risk score (PGRS) for bipolar disorder (BP)

Reference	Sample	fMRI Task	Contrast	Analysis	Association with PGRS / PGRS Threshold	Laterality	Brain Region	MNI coordinates
(Dima et al. 2016)	41 BP, 25 healthy relatives, and 46 HC White British ancestry Mean age: 40 Females: 48%	Facial Affect Recognition	Negative > neutral faces	Whole-brain *P* < 0.05 FWE correction and cluster size, k > 20	– / *P* = 0.05	L	Visual Association Cortex	−32, −88, 2
		Working Memory (N-back task)	2-back >baseline	Whole-brain P < 0.05 FWE correction and cluster size, k > 20	+ / *P* = 0.05	R	Medial Prefrontal Cortex	22, 48, −14
(Tesli et al. 2015)	85 BP and 121 HC Northern European Caucasians Mean age: 35 Females: 52%	Emotional Face Recognition	Negative faces > shapes	Whole-brain z-threshold = 2.3 cluster-level correction; result does not survive Bonferroni correction	+/ P = 0.05	R	Inferior Frontal Gyrus	52, 18, 12
			Negative > positive faces	Whole-brain z-threshold = 2.3 cluster-level correction; result does not survive Bonferroni correction	– / P = 0.05	R	Postcentral Gyrus	54, −20, 50
(Wang et al. 2017)	Dataset 1: 360 HC Dataset 2: 323 HC Chinese Mean Age: Dataset 1: 19.4 (SD = 1.1) Dataset 2: 22.7 (SD = 2.5) Females: Dataset 1: 48.3% Dataset 2: 51.4%	Resting state fMRI (functional connectivity)	NA	ROI voxel-level of P < 0.05 and cluster size >90 voxels to reach a cluster level significance of alpha <0.05	+ / *P* = 0.05	L	Cuneus with Insula	−9, −83, 31
					+ / P = 0.05	L	Precuneus with Insula	−3, −56, 7
					+ / P = 0.05	L	Posterior Cingulate Cortex with Insula	−12, −17, −23
					– / P = 0.05	R	Midbrain with Insula	12, −17, −20
(Whalley et al. 2012)	87 BP high risk and 71 HC Scottish Family Mean Age: BP high risk: 20.6 (SD = 2.4) HC: 20.89 (SD = 2.8) Females: BP high risk: 49% HC: 51%	Verbal Fluency (Hayling Sentence Completion Test)	Sentence completion > baseline	Whole-brain cluster level of P < 0.05, corrected for multiple comparisons	+ / P = 0.5	R	Anterior Cingulate Cortex	2, 40, −6
							Amygdala	18, −6, −14

*BP* Bipolar Disorder, *HC* Healthy Controls, *L* Left hemisphere, *NA* Not Applicable, *R* Right hemisphere, *ROI* Region of Interest

**Table 3:** Studies including the psychosis or cross-disorder polygenic risk score (PGRS)

Reference	Sample	PGRS	fMRI Task	Contrast	Analysis	Association with PGRS / PGRS Threshold	Laterality	Brain Region	MNI coordinates
(Lancaster et al. 2016b)	1528–1559 HC European Mean Age: 14.5 Females: 54%	Psychosis	Reward Processing (Monetary Incentive Task)	Reward anticipation	ROI P < .05, FWE	+ / P = 0.01; 0.05; 0.1; 0.5	L	Ventral Striatum	−12, 2, −8
									−18, 1, −8
									−18, 2, −5
				Reward receipt	ROI P < .05, FWE	+/ P = 0.01; 0.5	L	Ventral Striatum	−9, 5, −8
									−18, −1, −8
(Wang et al. 2017)	Dataset 1: 360 HC Dataset 2: 323 HC Chinese Mean Age: Dataset 1: 19.4 (SD = 1.1) Dataset 2: 22.7 (SD = 2.5) Females: Dataset 1: 48.3% Dataset 2: 51.4%	Cross-disorder	Resting state fMRI (functional connectivity)	NA	ROI voxel-level of P < 0.05 and cluster size >90 voxels to reach a cluster level significance of alpha <0.05	– / *P* = 0.05	L	Superior Temporal Gyrus with Insula	−57, −20, 7
								Supplementary Motor Area with Insula	−3, 4, 52
(Whalley et al. 2015)	82 BP high risk and 57 HC Scottish Family Mean Age: BP high risk: 21.1 (SD = 2.1) HC: 20.8 (SD = 2.3) Females: BP high risk: 45% HC: 51%	Cross-disorder	Verbal Fluency (Hayling Sentence Completion Test)	Sentence completion > baseline	Whole-brain cluster level P < 0.05, corrected for multiple comparisons	+ / P = 0.05 (driven by HC)	L	Inferior Frontal Gyrus, Precentral and Postcentral Gyri	−58, −14, 38

*BP* Bipolar Disorder, *HC* Healthy Controls, *L* Left hemisphere, *NA* Not Applicable, *R* Right hemisphere, *ROI* Region of Interest

#### Polygenic risk score for schizophrenia (PGRS-SCZ)

Of the 7 studies using PGRS for SCZ, six tested associations between PGRS and different fMRI tasks (four working memory, two emotional processing, one episodic memory, one theory of mind, one reward processing, one attention control processing, one probabilistic decision making) (Cosgrove et al. 2017; Erk et al. 2017; Kauppi et al. 2015; Lancaster et al. 2016a; Rampino et al. 2017; Walton et al. 2014), and one study reported results between PGRS-SCZ and resting state fMRI data (Wang et al. 2017). See Table 1 for details of these studies.

Of the four studies reporting results of working memory (WM), Kauppi et al. (2015) used a WM N-back task with a block design and 3 conditions (0-back, 2-back, and baseline) in 63 SCZ patients and 118 healthy controls, matched for education and IQ (mean > 100). Whole brain analysis was performed to explore whether increased genetic risk for SCZ would be associated with altered brain activation during WM processing. Ten PGRS-SCZ were initially computed based on different *p*-value thresholds for SNPs inclusion, however the threshold of 0.05 was chosen since it explained the most variance in case-control data. The PGRS-SCZ was negatively associated with brain activation during the 2-back >0-back contrast in the right inferior frontal gyrus, right middle/superior prefrontal cortex, and right middle temporal gyrus. The 2-back > baseline contrast revealed a negative correlation between PGRS and brain activation in the anterior cingulate cortex, right inferior frontal gyrus/insula, and in the bilateral postcentral gyrus. To correct for multiple comparisons the authors performed cluster-level correction with z-value >2.3 and a corrected cluster significance threshold of *P* = .05.

A positive association between PGRS calculated for SCZ and whole-brain neural activity during a WM block task was found in the study of Walton et al. (2014) in a sample of 92 SCZ patients and 114 healthy controls, matched for age, gender and parental education. For replication purposes, two independent discovery samples were used to calculate PGRS scores; from the International SCZ Consortium (ISC) (Purcell et al. 2009) and from the Psychiatric Genomics Consortium (PGC) (Ripke et al. 2011). Initially, the case-control dataset from the ISC were used to calculate PRGS using 7 different statistical thresholds (*P* < 0.01, *P* < 0.05, *P* < 0.1, *P* < 0.2, *P* < 0.3, *P* < 0.4, and *P* < 0.5). For a replication analysis, PGRS were computed using the PGC dataset following the method of Purcell and colleagues (P < 0.01) (Purcell et al. 2009). The Sternberg Item Recognition Paradigm (SIRP) was administered during six blocks composed of 1 (load 1), 3 (load 3), or 5 (load 5) digits. A positive relationship between PGRS_ISC (*P* < .01)_ and neural activity was found in the left dorsolateral prefrontal cortex (DLPFC) and left ventrolateral prefrontal cortex (VLPFC). Additionally, a positive association between neural activity in the anterior cingulate cortex (ACC) and bilateral DLPFC and PGRS _PGC (*P* < .01)_ was reported. All clusters were corrected with z-value >2.3 and Bonferroni corrected with a *P* value of .007 (.05/7). PGRS_ISC(*P* < .01)_ accounted for 4.3% of the total variance R^2^ = 0.048, *P* = .002 at the most activated DLPFC location (x, y, z: −6, 38, 48). A positive association was also found between the DLPFC and the PGRS-SCZ in the Walton et al. 2013 paper where for the same pool of participants PGRS-SCZ were calculated from 41 SNPs in 34 genes (SNPs were identified from a meta-analysis of genetic studies on schizophrenia available at www.schizophreniaresearchforum.org; February 24, 2010).

The study by Cosgrove et al. (2017) used a spatial WM task as well as a face processing task to identify association between cognitive performance and PGRS of 1016 genes whose expression is altered by miR-137 manipulation (Hill et al. 2014). It has been known that variants at microRNA-137 (MIR137) influence the expression on a set of genes, and some of them are independently associated with SCZ (Collins et al. 2014). 831 of these genes could be unambiguously mapped to the autosomes and this gene set was used to generate polygene scores. They then cross-referenced these genes with unweighted *P*-values from the PGC2 GWAS (Ripke et al. 2014) and a weighted polygene score was created for each participant. For the spatial block WM task three different conditions were used (baseline-, 1- and 3- dot task), and two contrasts investigated: spatial WM (1 dot and 3 dots vs baseline), and high spatial WM load (3 dots vs 1 dot). Analysis was performed at three different thresholds (*P* = 10^−5^, *P* = 0.05 and *P* = 0.5). Results were examined at a *P* < 0.001 (uncorrected) level and clusters were considered statistically significant at a *P* < 0.05 level, family-wise error (FWE) corrected for multiple comparisons across the whole brain at the cluster level. Results showed that higher PGRS-SCZ (P = 10^−5^) of the miR-137 pathway were significantly associated with increased neural activation in two clusters with increasing spatial WM load (3 dots vs 1 dot contrast). This hyper-activation was found in the right inferior occipital gyrus and right middle temporal gyrus. Cosgrove et al. (2017) also investigated associations between the PGRS-SCZ and fMRI activity during an emotional processing task including angry and neutral faces. For this task, no association was found significant.

Erk et al. (2017) tested five neuroimaging cognitive paradigms of the Research Domain Criteria (RDoC). PGRS-SCZ was calculated from summary data from the PGC (Ripke et al. 2014) to assess its effect on predefined regions of interest (ROIs) during WM, reward processing, episodic memory, social cognition and emotion processing tasks. Results for PGRS were significant only for the episodic memory task and social cognition (a Theory of Mind –ToM- task) at P_FWE (ROI)_ < 0.05, but would not withstand multiple comparison correction for the total number of ROI analyses performed across all tasks (*P* < 0.0025; 0.05/20 ROI analyses). For episodic memory 287 healthy individuals with no family history of SCZ and 195 healthy individuals with at least one first-degree relative affected by SCZ completed an associative episodic memory task requiring encoding, recall and recognition of face-profession pairs (a face linked to an occupation; memory>control). A significant negative correlation was found for pregenual anterior cingulate cortex activation and PGRS during episodic memory recognition. The association between ToM (mentalizing) processing and PGRS was tested in 281 healthy individuals without family history and 214 participants with family history of SCZ. A ROI analysis yielded that PGRS-SCZ predicted high activity in the posterior cingulate cortex and the precuneus during the ToM task (mentalizing>control), at P_FWE (ROI)_ < 0.05, but would not withstand multiple comparison correction for the total number of ROI analyses performed across all tasks (*P* < 0.0025; 0.05/20 ROI analyses).

Lancaster et al. (2016a) investigated the association between PGRS-SCZ and decision-making processing in 83 healthy controls with no history of psychiatric or neurological disorder and IQ score above 70. PGRS for SCZ was calculated at *P* < 0.5. Whole brain and ROI analyses were performed against choice (shift>stay) and outcome (reward>punishment) behavior. In the whole-brain analysis, a negative correlation was observed between activation of the right frontal pole and PGRS-SCZ in choice (P_FWE-WHOLEBRAIN_ corrected). In the ROI analysis of the choice processing an additional negative association was found between PGRS-SCZ and the left ventral striatum (P_FWE-ROI_ corrected). No correlation was found in the outcome condition.

The study by Rampino et al. (2017) used a Visual Attention Control (VAS) task to test the association between PGRS-SCZ as well as PGRS-SCZ_-Glu_ and brain activity in two independent samples of healthy participants consisting of 151 and 51 subjects respectively. Both the discovery and replication samples had no history of psychiatric or neurological conditions, of drug/alcohol abuse, or of trauma/loss of consciousness. PGRS-SCZ was calculated following the method reported by Purcell et al. (2009) with a threshold of *P* = 10^−8^. Subsequently, SNPs associated with glutamatergic neurotransmission pathway were detected along previously selected SNPs and PGRS-SCZ_Glu_ were calculated. The VAS task included three different load conditions with increasing level of attention. Results were thresholded at *P* < .05, small volume FWE corrected, by computing a mask of key regions of attention control processing. A significant positive correlation was found between the left superior frontal gyrus and PGRS-SCZ_Glu_ in both groups. No significant correlations were identified between PRS-SCZ and brain activity during the VAC task.

Lastly, Wang et al. (2017) inspected the effect of PGRS-SCZ on functional connectivity on fMRI resting state data on two independent groups of healthy participants, consisting of 360 and 323 individuals respectively. Analysis was based on the PGRS calculated at *P* < 0.05. Functional connectivity maps for each subject were calculated by computing correlation coefficients between time series in the insula (ROI bilateral) and time series from all other gray matter voxels throughout the brain. Age, sex, and the three top principle components were included in the regression model as covariates. Statistical maps were generated at a voxel-level of P < 0.05 and cluster size >90 voxels to reach a cluster level significance of alpha <0.05 (for multiple corrections the Alphasim algorithm was used). Altered functional connectivity was found with the bilateral insula and the left angular gyrus and the left dorsolateral prefrontal cortex associated with the PGRS-SCZ.

#### Polygenic risk score for bipolar disorder (PGRS-BP)

Of the four studies investigating the PGRS for BP, three analyzed associations between PGRS-BD and different fMRI tasks (one WM, two emotional face processing, one verbal fluency task) (Dima et al. 2016; Tesli et al. 2015; Whalley et al. 2012). Additionally, one study reported results using PGRS-BP and resting-state fMRI data (Wang et al. 2017). Details for these studies are presented in Table 2.

Dima et al. (2016) tested the PGRS-BD effect on WM and emotional face recognition using fMRI data in 41 affected patients, 25 healthy first degree relatives and 46 healthy controls. PGRS for BD were based on BD summary statistics from the latest PGC GWAS of BD (Cross-Disorder Group of the Psychiatric Genomics Consortium 2013). PGRS-BD scores were derived using seven statistical thresholds with *P*-values <0.001, 0.05, 0.1, 0.2, 0.3, 0.4 and 0.5. Although the effect of group was significant at all thresholds, the highest difference (F = 5.94, *p* = 0.004) was found for the PGR-BD score with *P* < 0.1, thus this threshold was chosen for further analysis. A negative association between PGRS-BD and emotional face recognition was found in the visual cortex, and a positive association in the ventromedial prefrontal cortex for the WM task (*P* < 0.05 FWE correction and cluster size, k > 20).

In the study by Tesli et al. (2015), 85 bipolar patients and 121 healthy controls participated in an fMRI emotional faces matching paradigm with four different conditions: negative faces > shapes, positive faces > shapes, faces > shapes, and positive > negative faces. A total of ten PGRS were computed for BD (calculated using summary statistics by Sklar et al. 2011) based on different *P*-value thresholds (*P* = 1, 0.5, 0.4, 0.3, 0.2, 0.1, 0.05, 0.01, 0.001, and 0.0001). The PGRS explaining most of the variance patients vs controls was the one with a P-value threshold of 0.05. A positive correlation between PGRS-BD and brain activation was found in the right inferior frontal gyrus (negative > shapes), and a negative association was found in the right postcentral gyrus (negative > positive) across the whole group, z-threshold>2.3 as cluster-level correction although neither result survived a Bonferroni correction.

Whalley et al. (2012) investigated the relationship between PGRS-BD (calculated using summary statistics by Sklar et al. 2011) and brain activation during an fMRI verbal fluency task (the Hayling Sentence Completion Test) in 87 individuals with high risk for BD and 71 healthy controls. In total, 4 PGRS-BD were computed (*P* = 0.001, *P* = 0.01, *P* = 0.05, *P* = 0.1); the *P* = 0.5 was chosen for subsequent analysis as this level most efficiently discriminated individuals with and without BD. The whole brain analysis revealed a significant positive association between PGRS-BD and activation in the anterior cingulate cortex and in the right amygdala with increasing task difficulty (significant at a cluster level of *P* < 0.05, corrected for multiple comparisons).

The study by Wang et al. (2017) tested the PGRS-BP (calculated from the Cross-Disorder Group of the Psychiatric Genomics Consortium 2013 at P < 0.05) on functional connectivity of resting state fMRI data. A significant positive relationship was found between PGRS-BP and functional connectivity between the insula (ROI insula) and the bilateral cuneus, precuneus and posterior cingulate. A negative relationship was identified for the PGRS-BD and functional connectivity between the bilateral midbrains and the bilateral insula (cluster level significance of alpha <0.05). Age, sex, and the three top principle components were included in the regression model as covariates.

#### Cross-disorder and psychosis polygenic risk scores

Investigating PGRSs for cross-disorder psychopathology (PGRS-CROSS) is justified since evidence has revealed shared genetic links across five common psychiatric illnesses (autism, attention deficit-hyperactivity disorder, BD, major depressive disorder and SCZ) based on accumulated effects of many common variants (Cross-Disorder Group of the Psychiatric Genomics Consortium 2013). Two studies examined the influence of PGRS-CROSS (Wang et al. 2017; Whalley et al. 2015) and one study the influence of PGRS for psychosis (Lancaster et al. 2016b) on brain function and were included in the present systematic review (Table 3). Studies examining PGRS for psychosis utilize the fact that psychotic disorders share considerable genetic variance (Ruderfer et al. 2014), have substantial overlap in the clinical phenotype (Craddock et al. 2009) and have shown brain activation changes in similar frontal networks (Birur et al. 2017). This stream of research tests the hypothesis that SCZ and BD lie on a transdiagnostic psychosis spectrum with overlapping affective and non-affective psychotic symptoms (Reininghaus et al. 2016).

In the Lancaster et al. (2016b) study, PGRS for psychosis was calculated for healthy adolescents using PGC summary statistics for SCZ and BD (Ruderfer et al. 2014). Just over 1500 participants performed an fMRI task evaluating reward processing during two conditions; reward anticipation and reward receipt. PGRS scores were derived using four thresholds with *P*-values <0.01, 0.05, 0.1 and 0.5. Only the association with the ventral striatum was examined, with age, sex, testing site, IQ and the first five principle components (from the variance-standardized relationship matrix of the linkage disequilibrium–pruned genotypes) were included in the regression models. Analysis revealed a significant positive association between PGRS for psychosis and activation in the left ventral striatum in anticipation for all psychosis PGRS thresholds and in reward receipt for PGRS thresholds, *P* < 0.01 and 0.5. The results survived multiple testing using FWE correction (*P* < .05). Additionally, a post-hoc analysis investigating the PGRS for BD and SCZ separately found that activity in the ventral striatum during reward processing was influenced by both scores.

Whalley et al. (2015) tested the effect of the PGRS-CROSS (Cross-Disorder Group of the Psychiatric Genomics Consortium 2013; significance level of *P* = 0.05) in a group of 82 people with family risk for BD and 57 healthy controls during an fMRI verbal fluency task (the Hayling Sentence Completion Test). There were no significant associations across the whole sample, but a significant interaction was found between PGRS-CROSS and group status in the frontal cortex encompassing the left inferior frontal gyrus, precentral and postcentral gyri. This interaction was driven by healthy controls, and not by individuals at high risk of bipolar disorder. Although, statistical maps were thresholded at a level of *P* < 0.001 (uncorrected), regions reported as significant survived at a cluster level of *P* < 0.05, corrected for multiple comparisons. Further analyses revealed that the PGRS contributing the greatest effect was specific to SCZ.

Wang et al. (2017) investigated the effect of PGRS-CROSS on functional connectivity in fMRI resting state data. GWAS results from the PGC (Cross-Disorder Group of the Psychiatric Genomics Consortium 2013) were used as training data to generate PRGS-CROSS. In two independent datasets, with 360 and 323 healthy participants respectively, functional connectivity maps for each subject were calculated by computing correlation coefficients between time series in the ROI (bilateral insula) and time series from all other gray matter voxels throughout the brain. A negative relationship was identified between the PGRS-CROSS and the coupling of the left supplementary motor and left superior temporal gyrus with bilateral insula.

## Discussion

This is the first systematic review to examine the effects of PGRS-SCZ, PGRS-BD, PGRS-CROSS and PGRS for psychosis on brain function and connectivity using fMRI data. The diversity of designs, tasks, and types of measurement precludes us from performing a meta-analysis, but allows us to summarize the findings and draw some conclusions.

### PGRS and memory

The results of four studies focusing on WM-related brain activation and the effect of PGRS-SCZ on it demonstrated that: (a) healthy individuals with high PGRS-SCZ showed increased cortical activation in the right inferior occipital gyrus and the right middle temporal gyrus (Cosgrove et al. 2017), (b) there is a negative relationship between PGRS-SCZ and inferior/middle prefrontal cortex and middle temporal gyrus activation (Kauppi et al. 2015), (c) there is a positive association between PGRS-SCZ and WM-related brain activation in the left dorsolateral and ventrolateral prefrontal cortex (Walton et al. 2014) and (d) no association was found in the Erk et al. (2017) study between WM brain activation and PGRS-SCZ.

The hyper-activation found in the right inferior occipital gyrus and right middle temporal gyrus in individuals with high miR-137 PGRS might reflect relative cortical inefficiency which is associated with schizophrenia (Cosgrove et al. 2017). Although Kauppi et al. (2015) and Walton et al. (2014) used the same method to calculate PGRS-SCZ to test WM-related brain activation, the results are contradictory showing both frontal hyper- and hypo-activation during WM processing. One possible source of difference between results of these studies is that similar but not identical WM tasks were used as well as the contrasts tested against the PGRS. The Sternberg Item Recognition Paradigm used by Walton et al. (2014) emphasizes the maintenance of information, while the N-back WM task used by Kauppi et al. (2015) accentuates updating processes. Another reason for the contradictory findings could be brain activation differences between high and low WM load conditions in individuals with high PGRS-SCZ. There might be a positive relationship between PGRS-SCZ and brain activation at low WM loads reflecting cognitive effort, while hypo-activation of frontal lobe regions at more demanding tasks could be explained due to neural inefficiency and decreased flexibility in recruiting neural resources in response to task difficulty (Kauppi et al. 2015; Kim et al. 2010; Potkin et al. 2009). It has also been argued that there is a complex pattern of hypo- and hyperactivation across the brain in response to WM processing in SCZ, and that rather than looking at individual brain regions, the entire network of activation and co-activation needs to be taken into account (Glahn et al. 2005). This might also be true for individuals with high PGRS for SCZ, reflected in the differences in findings reported across studies reviewed here, and speaking to the need for multivariate analyses methods in future studies.

When it comes to WM processing in BP, Dima et al. (2016) found hyper-activation of the frontal lobe (particularly the ventromedial prefrontal cortex) in individuals with high PGRS for BD, although the areas of abnormal activation differed from studies of the PGRS for SCZ. The ventromedial prefrontal cortex integrates information which is represented in separate parts of the limbic system (the hippocampus, the amygdala, and the ventral striatum), and its function is reduced during cognitive tasks performance (Nieuwenhuis and Takashima 2011). However, people with high genetic risk for BD failed to deactivate this area during a memory task and instead demonstrated hyper-activation (Dima et al. 2016).

Investigating the relationship between PGRS-SCZ and episodic memory processing, Erk et al. (2017) found that the PGRS-SCZ is associated with hypo-activation of the pregenual anterior cingulate cortex during this task. Many studies have shown evidence of episodic memory dysfunction in SCZ (Leavitt and Goldberg 2009; Ranganath et al. 2008), and this has been associated with prefrontal activation deficits (Achim and Lepage 2005). This may point to the reason why individuals with high PGRS for SCZ demonstrated hypoactivation in left anterior cingulate cortex during episodic memory recognition in the study by Erk et al. (2017).

### PGRS and emotional processing

BD is characterized by disturbances in emotional processing and regulation (Green et al. 2007; Townsend and Altshuler 2012), that are likely influenced by genetic factors (Brotman et al. 2008; Lelli-Chiesa et al. 2011; Radua et al. 2012). Altered brain activation during emotional processing in relation to PGRS-BD were explored in two studies reviewed here. Functional abnormalities were found (a) in the left visual association cortex during negative vs neutral face recognition (Dima et al. 2016), and (b) in the right inferior frontal gyrus during negative faces vs shapes and in the right postcentral gyrus during negative vs positive face processing (Tesli et al. 2015). These findings are in line with the hypothesis that BD is caused by a combination of interacting genetic factors that are associated with abnormalities of emotional processing.

Erk et al. (2017) and Cosgrove et al. (2017) both investigated links between the PGRS for SCZ and emotional processing, but neither found evidence of an association. Emotional processing has been found to be impaired in patients with SCZ (Bora and Pantelis 2016; Edwards et al. 2002; Marwick and Hall 2008) as well as in unaffected relatives of SCZ patients (Bediou et al. 2007; Lavoie et al. 2013; Park et al. 2016), suggesting a genetic component to this disturbance. The lack of significant associations between genetic risk for SCZ and brain activation during emotional processing in the two studies reviewed here (Cosgrove et al. 2017; Erk et al. 2017) could be because the PGRS-SCZ is picking up primarily working memory disturbances, but as discovery sample sizes continue to grow and the PGRS-SCZ becomes more sensitive, emotional areas might be identified too. Alternatively, associations between emotional brain activation and genetic risk for SCZ might be driven by genetic markers not captured by the current PGRS, such are rare variants.

### PGRS and other cognitive processes

Investigating the relationship between PGRS-SCZ and ToM processing Erk et al. (2017) found a positive correlation between PGRS-SCZ and activity in the posterior cingulate cortex and the precuneus complex during mentalizing processing. Mentalizing is an important social cognition function that refers to the ability to understand one’s own and other peoples’ mental states. There is evidence that people with SCZ are impaired in mentalizing and ToM performance (Bora et al. 2009) and previous work has linked genetic risk alleles associated with schizophrenia (in the ZNF804A gene) with decreasing activity of the left temporo-parietal junction, dorsomedial prefrontal cortex and the posterior cingulate cortex, during ToM processing in healthy individuals (Mohnke et al. 2014; Walter et al. 2011). Furthermore, there is evidence that a number of genetic markers relating to ToM impairment are associated not only with schizophrenia, but across several psychiatric conditions (Martin et al. 2014). It would therefore be useful for future research to address the genetic associations with ToM processing across diagnostic boundaries.

Lancaster et al. (2016a) investigated the association between PGRS-SCZ and brain activity during decision making processing. They found a negative association between PGR-SCZ and right frontal pole and the left ventral striatum activation. Hypo-activation of the right frontal pole has previously been associated with cognitive deficit symptoms of SCZ (Orellana and Slachevsky 2013). The current results demonstrate a negative relationship between PGRS-SCZ and frontostriatal activation and may reflect cumulative genetic susceptibility for SCZ affecting parameters of probabilistic learning. Additionally, frontal hypoactivation may be linked with poor motivation associated with negative symptoms of SCZ and these changes in neural networks may reflect genetic risk for this disorder (Lancaster et al. 2016a).

A recent meta-analysis of 23 studies revealed that psychosis is associated with hypoactivation of the ventral striatum during reward anticipation in adults (Radua et al. 2015). However, in a large sample of healthy adolescents, Lancaster et al. (2016b) found that increased polygenetic risk for psychosis is associated with hyper-activation of this brain region during reward processing. This difference in findings could be – as suggested by Lancaster et al. (2016b) – due to the difference in age between the samples, and genetic risk for psychosis might have different effects on brain function across the lifespan. It has, for example, been shown that younger adolescent offspring of patients with schizophrenia show hyper-activation, whereas older adolescent offspring show hypo-activation, of the ventral striatum during reward processing (Vink et al. 2016). Recent studies have demonstrated the significant role of methylation quantitative trait loci in brain development and SCZ (Hannon et al. 2016; Jaffe et al. 2016) as well as significant associations between methylation and gray matter and SCZ diagnosis in both saliva and blood tissues (Lin et al. 2018). Epigenetics do play a role and future studies should combine epigenomic/genomic analyses in schizophrenia cases to decipher how genetic risks influence structural and functional brain components through epigenetic mediation.

Several neuroimaging studies have identified modification in prefrontal cortex processing of attention stimuli in patients with schizophrenia (Blasi et al. 2010; Delawalla et al. 2008). There is evidence that variation in attention is partially associated to glutamatergic neurotransmission pathways (Craven et al. 2014). Investigating the relationship between polygenic risk related to glutamatergic neurotransmission and attention control processing, Rampino et al. (2017) found a positive association between PGRS-SCZ_Glu_ and prefrontal cortex activity during the VAC sustained attention task. The results show that greater engagement of prefrontal resources leads to lower performance efficiency in people with high genetic risk for schizophrenia during VAC task. Additionally, no relationship between PGRS-SCZ and fMRI activity was found suggesting that pathway specific PGRS may be a more useful tool in detecting genes-brain-behavior relationships.

In two studies, Whalley and colleagues investigated associations between brain activation during a language-based executive processing task and the PGRS-BP (Whalley et al. 2012) and the cross-disorder PGRS (Whalley et al. 2015). They found a significant positive association between PGRS-BD and activation of the anterior cingulate cortex and amygdala (Whalley et al. 2012). The existing literature on imaging studies of BD provides evidence for the involvement of these brain regions (Arnone et al. 2009; Chen et al. 2011; Strakowski et al. 2012) and results from the study by Whalley et al. (2012) indicate that the effect of genetic load for BD on brain function affects task-related recruitment of these brain regions. There was also a positive association between the cross-disorder PGRS and brain activation during executive functioning in frontal regions, driven by genetic risk for SCZ (Whalley et al. 2015). Again, this is consistent with previous research implicating altered frontal activation in SCZ during executive function tasks (Costafreda et al. 2011; Sutcliffe et al. 2016).

Lastly, Wang et al. (2017) explored the relationship between resting state functional connectivity and cross-disorder PGRS as well as PGRS for specific disorders in healthy individuals. Results showed that altered bilateral insular functional connectivity patterns correlated with increased cross-disorder as well as PGRS- SCZ and BP, indicating that such functional connectivity changes might be genetically modulated (Wang et al. 2017). The insula is a key node in the salience network that is important for detecting behaviorally relevant stimuli, and altered processing of the insula is implicated in a range of major psychiatric illnesses including SCZ and BP (Uddin 2014; White et al. 2010). Altered functional connectivity between the insula and the posterior cingulate cortex and the precuneus was associated with the PGRS for BP, and altered insula – angular gyrus connectivity was associated with the PGRS for SCZ (Wang et al. 2017). These regions are part of the default mode network (mainly active whilst not performing a cognitive task), which has previously been implicated in these disorders (Öngür et al. 2010). The PGRS for SCZ was furthermore associated with altered connectivity between the insula and the dorsolateral prefrontal cortex (Wang et al. 2017), which is a hub of the central-executive network that show increased activity during cognitively demanding tasks (Sridharan et al. 2008). It is thought that the salience network plays an important role in switching between the default-mode and the central-executive networks (Menon and Uddin 2010; Sridharan et al. 2008) and results reviewed here might indicate that a dysfunction of this switching mechanism is mediated by genetic risk for psychiatric illnesses including SCZ and BP.

### Limitations, future directions and conclusions

The conclusions from this systematic review should be considered in the light of the following limitations in the literature. First, most studies used small sample sizes – with the notable exception of Lancaster et al. (2016b) including over 1500 individuals – limiting the possibility of drawing any strong conclusions from findings (for more detail on appropriate sample sizes see Dudbridge 2013; Visscher et al. 2017). Secondly, there are significant differences in methodology, making it difficult to compare findings across studies. This involves (i) the inclusion of different combinations of healthy individuals, people at high genetic risk for psychiatric disorders, and patients themselves, (ii) important differences in fMRI analyses methods, with some restricting their analyses to regional effects using a ROI approach, whereas others use whole-brain analyses, (iii) the use of different *P*-value thresholds for the calculations of PGRS and (iv) only two studies (Cosgrove et al. 2017; Rampino et al. 2017) utilize the PGC SCZ summary statistics by considering a specific biological pathway (the miR-137 pathway; glutamatergic neurotransmission pathway). Since it is unlikely that the combined effect of all genetic markers for a disorder should influence a single neural circuit, exploring biological pathways when conducting these types of analyses might result in more informative findings (Hall et al. 2015). Lastly, most studies did not investigate possible confounding factors that might influence results, such as environmental risk factors.

To limit the impact of these issues and for this field of research to move forward and produce more conclusive findings, we propose the following recommendations: 1) an increase in the sample sizes of studies, through collaborations and the development of consortia, 2) replication of existing findings, 3) the development of optimum PGRS thresholding approaches (e.g. choosing the PGRS that best predicts the target data phenotype, to accommodate the fact that the discovery summary statistics GWAS data can vary by p-threshold depending on heritability, sample size or by applying high-resolution PGRS detection – Euesden et al. 2015), 4) standardization of fMRI paradigms, 5) collecting a resting state fMRI which is not confounded by differences in performance as part of acquisition pipelines, 6) correlating brain activity from fMRI tasks with a standardized psychometric test battery collected outside of the scanner that are potentially scalable online to large cohorts common in genetic studies, 7) the use of data reduction and multivariate machine learning methods to investigate networks of brain co-activation rather than focusing on individual regions, 8) studies to explore PGRS for specific biological pathways in imaging studies to elucidate neurobiological mechanisms in order to further the understanding of underlying mechanisms and to develop new hypotheses and 9) reporting of effect sizes to facilitate comparison across studies.

Furthermore, limitation (ii) regarding differences in fMRI analyses methods, is a challenge faced not only by the imaging genetics field but by the wider fMRI neuroimaging community and has recently been commented by Poldrack et al. (2017) that have proposed some excellent solutions. Briefly, the authors propose (a) planning (sample size should be pre-determined using power analysis and the analysis plan pre-registered), (b) implementation (codes for data collection and analysis, and data sets and results should be placed in a repository), (c) validation (exploratory results should be validated against an independent validation data set) and (d) dissemination (results should be marked as either hypothesis-driven, with a link to pre-registration, or exploratory. All analyses performed on the data, significant, useful or not should be reported) (Poldrack et al. 2017).

In the last years, advances have been made in large-scale neuroimaging genetics studies, such as the innovative Alzheimer’s Disease Neuroimaging Initiative (ADNI; Weiner et al. 2010) and the ENIGMA consortium which took the novel approach by combining existing genomic and imaging data from around the globe (Bearden and Thompson 2017). Current big data initiatives, like the UK Biobank a prospective epidemiological resource gathering extensive questionnaires, physical measures, cognitive measures, neuroimaging and genotyping data in a cohort of 500,000 participants has minimized variations in scanner, acquisition and analytical approaches (Sudlow et al. 2015) and holds great promise for the future.

In conclusion, the existing literature has important methodological limitations, but has nevertheless provided some preliminary findings regarding the relationships between brain activation and cumulative genetic risk for SCZ and BD. Overall, it suggests that the effect of PGRS for SCZ or BD is not localized to a single region or neuronal pathway, but instead influence task-dependent brain activation of whole brain networks. The current findings do however support the notion that the PGRS methodology could be informative in terms of identifying patterns of neural activation that could mediate vulnerability to SCZ or BD rather than symptom expression. Future imaging genetics studies with large samples would be uniquely informative in mapping the spatial distribution of the genetic risk to psychiatric disorders on brain processes during various cognitive tasks and may lead to the discovery of biological pathways that may be crucial in mediating the link between genetic factors and alterations in brain networks in this disorder. As interest in imaging genetics research continues to grow, multimodal neuroimaging and PGRS approaches are likely to become major tools in the investigation of the pathophysiology of psychiatric disorders.

## References

[CR1] Achim AM, Lepage M (2005). Episodic memory-related activation in schizophrenia: meta-analysis. The British Journal of Psychiatry.

[CR2] Arnone D, Cavanagh J, Gerber D, Lawrie SM, Ebmeier KP, McIntosh AM (2009). Magnetic resonance imaging studies in bipolar disorder and schizophrenia: Meta-analysis. The British Journal of Psychiatry.

[CR3] Bearden CE, Thompson PM (2017). Emerging Global Initiatives in Neurogenetics: The Enhancing Neuroimaging Genetics through Meta-analysis (ENIGMA) Consortium. Neuron.

[CR4] Bediou B, Asri F, Brunelin J, Krolak-Salmon P, D’Amato T, Saoud M (2007). Emotion recognition and genetic vulnerability to schizophrenia. The British Journal of Psychiatry.

[CR5] Bigos KL, Weinberger DR (2010). Imaging genetics-days of future past. NeuroImage.

[CR6] Birur B, Kraguljac NV, Shelton RC, Lahti AC (2017). Brain structure, function, and neurochemistry in schizophrenia and bipolar disorder-a systematic review of the magnetic resonance neuroimaging literature. NPJ Schizophrenia.

[CR7] Blasi G, Taurisano P, Papazacharias A, Caforio G, Romano R, Lobianco L (2010). Nonlinear response of the anterior cingulate and prefrontal cortex in schizophrenia as a function of variable attentional control. Cerebral Cortex.

[CR8] Bogdan R, Salmeron BJ, Carey CE, Agrawal A, Calhoun VD, Garavan H, Hariri AR, Heinz A, Hill MN, Holmes A, Kalin NH, Goldman D (2017). Imaging Genetics and Genomics in Psychiatry: A Critical Review of Progress and Potential. Biological Psychiatry.

[CR9] Bora E, Pantelis C (2016). Social cognition in schizophrenia in comparison to bipolar disorder: A meta-analysis. Schizophrenia Research.

[CR10] Bora E, Yucel M, Pantelis C (2009). Theory of mind impairment in schizophrenia: Meta-analysis. Schizophrenia Research.

[CR11] Brotman MA, Guyer AE, Lawson ES, Horsey SE, Rich BA, Dickstein DP (2008). Facial emotion labeling deficits in children and adolescents at risk for bipolar disorder. The American Journal of Psychiatry.

[CR12] Cardno AG, Marshall EJ, Coid B, Macdonald AM, Ribchester TR, Davies NJ (1999). Heritability estimates for psychotic disorders: the Maudsley twin psychosis series. Archives of General Psychiatry.

[CR13] Chen CH, Suckling J, Lennox BR, Ooi C, Bullmore ETA (2011). quantitative meta-analysis of fMRI studies in bipolar disorder. Bipolar Disorders.

[CR14] Collins AL, Kim Y, Bloom RJ, Kelada SN, Sethupathy P, Sullivan PF (2014). Transcriptional targets of the schizophrenia risk gene MIR137. Translational Psychiatry.

[CR15] Cosgrove D, Harold D, Mothersill O, Anney R, Hill MJ, Bray NJ (2017). MiR-137-derived polygenic risk: effects on cognitive performance in patients with schizophrenia and controls. Translational Psychiatry.

[CR16] Costafreda SG, Fu CHY, Picchioni M, Toulopoulou T, McDonald C, Kravariti E (2011). Pattern of neural responses to verbal fluency shows diagnostic specificity for schizophrenia and bipolar disorder. BMC Psychiatry.

[CR17] Craddock N, O’Donovan MC, Owen MJ (2009). Psychosis genetics: modeling the relationship between schizophrenia, bipolar disorder, and mixed (or “schizoaffective”) psychoses. Schizophrenia Bulletin.

[CR18] Craven AR, Johnsen E, Kroken RA, Falkenberg LE (2014). Impact of glutamate levels on neuronal response and cognitive abilities in schizophrenia. NeuroImage: Clinical.

[CR19] Cross-Disorder Group of the Psychiatric Genomics Consortium (2013). Identification of risk loci with shared effects on five major psychiatric disorders: a genome-wide analysis. Lancet.

[CR20] Delawalla Z, Csernansky JG, Barch DM (2008). Prefrontal cortex function in nonpsychotic siblings of individuals with schizophrenia. Biological Psychiatry.

[CR21] Dima D, Breen G (2015). Polygenic risk scores in imaging genetics: Usefulness and applications. Journal of Psychopharmacology.

[CR22] Dima D, de Jong S, Breen G, Frangou S (2016). The polygenic risk for bipolar disorder influences brain regional function relating to visual and default state processing of emotional information. NeuroImage: Clinical.

[CR23] Dudbridge F (2013). Power and Predictive Accuracy of Polygenic Risk Scores. PLoS Genetics.

[CR24] Edwards J, Jackson HJ, Pattison PE (2002). Emotion recognition via facial expression and affective prosody in schizophrenia: A methodological review. Clinical Psychology Review.

[CR25] Elliott, L., Sharp, K., Alfaro-Almagro, F., Douaud, G., Miller, K., Marchini, J., & Smith, S. (2017). The genetic basis of human brain structure and function: 1,262 genome-wide associations found from 3,144 GWAS of multimodal brain imaging phenotypes from 9,707 UK Biobank participants. *bioRxiv*, 178806. 10.1101/178806.

[CR26] Erk S, Meyer-Lindenberg A, Schmierer P (2013). Hippocampal and frontolimbic function as intermediate phenotype for psychosis: Evidence from healthy relatives and a common risk variant in CAC- NA1C. Biological Psychiatry.

[CR27] Erk S, Mohnke S, Ripke S, Lett TA, Veer IM, Wackerhagen C (2017). Functional neuroimaging effects of recently discovered genetic risk loci for schizophrenia and polygenic risk profile in five RDoC subdomains. Translational Psychiatry.

[CR28] Euesden J, Lewis CM, O'Reilly PF (2015). PRSice: Polygenic Risk Score software. Bioinformatics.

[CR29] Farrell MS, Werge T, Sklar P, Owen MJ, Ophoff RA, O’Donovan MC (2015). Evaluating historical candidate genes for schizophrenia. Molecular Psychiatry.

[CR30] Geschwind DH, Flint J (2015). Genetics and genomics of psychiatric disease. Science (80-. ).

[CR31] Glahn DC, Ragland JD, Abramoff A, Barrett J, Laird AR, Bearden CE, Velligan DI (2005). Beyond hypofrontality: A quantitative meta-analysis of functional neuroimaging studies of working memory in schizophrenia. Human Brain Mapping.

[CR32] Green MJ, Cahill CM, Malhi GS (2007). The cognitive and neurophysiological basis of emotion dysregulation in bipolar disorder. Journal of Affective Disorders.

[CR33] Hall J, Trent S, Thomas KL, O'Donovan MC, Owen MJ (2015). Genetic risk for schizophrenia: convergence on synaptic pathways involved in plasticity. Biological Psychiatry.

[CR34] Hannon E, Spiers H, Viana J (2016). Methylation QTLs in the developing brain and their enrichment in schizophrenia risk loci. Nature Neuroscience.

[CR35] Hashimoto R, Ohi K, Yamamori H, Yasuda Y, Fujimoto M, Umeda-Yano S (2015). Imaging genetics and psychiatric disorders. Current Molecular Medicine.

[CR36] Hibar DP, Stein JL, Renteria ME (2015). Common genetic variants influence human subcortical brain structures. Nature.

[CR37] Hibar DP, Adams HHH, Jahanshad N, Chauhan G, Stein JL, Hofer E (2017). Novel genetic loci associated with hippocampal volume. Nature Communications.

[CR38] Hill MJ, Donocik JG, Nuamah RA, Mein CA, Sainz-Fuertes R, Bray NJ (2014). Transcriptional consequences of schizophrenia candidate miR-137 manipulation in human neural progenitor cells. Schizophrenia Research.

[CR39] Jaffe AE, Gao Y, Deep-Soboslay A (2016). Mapping DNA methylation across development, genotype and schizophreniain the human frontal cortex. Nature Neuroscience.

[CR40] Kauppi K, Westlye LT, Tesli M, Bettella F, Brandt CL, Mattingsdal M (2015). Polygenic risk for schizophrenia associated with working memory-related prefrontal brain activation in patients with schizophrenia and healthy controls. Schizophrenia Bulletin.

[CR41] Kerner B (2015). Toward a deeper understanding of the genetics of bipolar disorder. Frontiers in Psychiatry.

[CR42] Kieseppa T, Partonen T, Haukka J, Kaprio J, Lonnqvist J (2004). High concordance of bipolar I disorder in a nationwide sample of twins. American Journal of Psychiatry.

[CR43] Kim MA, Tura E, Potkin SG, Fallon JH, Manoach DS, Calhoun VD (2010). Working memory circuitry in schizophrenia shows widespread cortical inefficiency and compensation. Schizophrenia Research.

[CR44] Lancaster TM, Ihssen N, Brindley LM, Tansey KE, Mantripragada K, O’Donovan MC (2016). Associations between polygenic risk for schizophrenia and brain function during probabilistic learning in healthy individuals. Human Brain Mapping.

[CR45] Lancaster TM, Linden DE, Tansey KE, Banaschewski T, Bokde ALW, Bromberg U (2016). Polygenic Risk of Psychosis and Ventral Striatal Activation During Reward Processing in Healthy Adolescents. JAMA Psychiatry.

[CR46] Lavoie MA, Plana I, Bédard Lacroix J, Godmaire-Duhaime F, Jackson PL, Achim AM (2013). Social cognition in first-degree relatives of people with schizophrenia: A meta-analysis. Psychiatry Research.

[CR47] Leavitt VM, Goldberg TE (2009). Episodic memory in schizophrenia. Neuropsychology Review.

[CR48] Lelli-Chiesa G, Kempton MJ, Jogia J, Tatarelli R, Girardi P, Powell J (2011). The impact of the Val158Met catechol- O-methyltransferase genotype on neural correlates of sad facial affect processing in patients with bipolar disorder and their relatives. Psychological Medicine.

[CR49] Lichtenstein P, Yip BH, Björk C, Pawitan Y, Cannon TD, Sullivan PF (2009). Common genetic influences for schizophrenia and bipolar disorder: A population-based study of 2 million nuclear families. Lancet.

[CR50] Lin D, Chen J, Ehrlich S, Bustillo JR, Perrone-Bizzozero N, Walton E (2018). Cross-Tissue Exploration of Genetic and Epigenetic Effects on Brain Gray Matter in Schizophrenia. Schizophrenia Bulletin.

[CR51] Martin AK, Robinson G, Dzafic I, Reutens D, Mowry B (2014). Theory of mind and the social brain: Implications for understanding the genetic basis of schizophrenia. Genes, Brain Behavior.

[CR52] Marwick K, Hall J (2008). Social cognition in schizophrenia: A review of face processing. British Medical Bulletin.

[CR53] Menon V, Uddin LQ (2010). Saliency, switching, attention and control: a network model of insula function. Brain Structure & Function.

[CR54] Moher D, Shamseer L, Clarke M, Ghersi D, Liberati A, Petticrew M (2015). Preferred reporting items for systematic review and meta-analysis protocols (PRISMA-P) 2015 statement. Systematic Reviews.

[CR55] Mohnke S, Erk S, Schnell K, Schütz C, Romanczuk-Seiferth N, Grimm O (2014). Further Evidence for the Impact of a Genome-Wide-Supported Psychosis Risk Variant in ZNF804A on the Theory of Mind Network. Neuropsychopharmacology.

[CR56] Mufford MS, Stein DJ, Dalvie S, Groenewold NA, Thompson PM, Jahanshad N (2017). Neuroimaging genomics in psychiatry-a translational approach. Genome Medicine.

[CR57] Mühleisen TW, Leber M, Schulze TG, Strohmaier J, Degenhardt F, Treutlein J (2014). Genome-wide association study reveals two new risk loci for bipolar disorder. Nature Communications.

[CR58] Nieuwenhuis ILC, Takashima A (2011). The role of the ventromedial prefrontal cortex in memory consolidation. Behavioural Brain Research.

[CR59] Öngür D, Lundy M, Greenhouse I, Shinn AK, Menon V, Cohen BM (2010). Default mode network abnormalities in bipolar disorder and schizophrenia. Psychiatry Research - Neuroimaging.

[CR60] Orellana G, Slachevsky A (2013). Executive functioning in schizophrenia. Frontiers in Psychiatry.

[CR61] Park HY, Yun J-Y, Shin NY, Kim S-Y, Jung WH, Shin YS (2016). Decreased neural response for facial emotion processing in subjects with high genetic load for schizophrenia. Progress in Neuro-Psychopharmacology & Biological Psychiatry.

[CR62] Paulus FM, Bedenbender J, Krach S (2014). Association of rs1006737 in CACNA1C with alterations in prefrontal activation and fronto-hippocampal connectivity. Human Brain Mapping.

[CR63] Pezawas L, Meyer-Lindenberg A (2010). Imaging genetics: Progressing by leaps and bounds. NeuroImage.

[CR64] Poldrack RA, Baker CI, Durnez J, Gorgolewski KJ, Matthews PM, Munafò MR, Nichols TE (2017). Scanning the horizon: towards transparent and reproducible neuroimaging research. Nature Reviews. Neuroscience.

[CR65] Potkin SG, Turner JA, Brown GG, McCarthy G, Greve DN, Glover GH (2009). Working memory and DLPFC inefficiency in schizophrenia: The FBIRN study. Schizophrenia Bulletin.

[CR66] Purcell SM, Wray NR, Stone JL, Visscher PM, O’Donovan MC, Sullivan PF (2009). Common polygenic variation contributes to risk of schizophrenia and bipolar disorder. Nature.

[CR67] Radua J, Surguladze SA, Marshall N, Walshe M, Bramon E, Collier DA (2012). The impact of CACNA1C allelic variation on effective connectivity during emotional processing in bipolar disorder. Molecular Psychiatry.

[CR68] Radua J, Schmidt A, Borgwardt S, Heinz A, Schlagenhauf F, McGuire P (2015). Ventral Striatal Activation During Reward Processing in Psychosis. JAMA Psychiatry.

[CR69] Rampino A, Taurisano P, Fanelli G, Attrotto M, Torretta S, Antonella L (2017). A Polygenic Risk Score of glutamatergic SNPs associated with schizophrenia predicts attentional behavior and related brain activity in healthy humans. European Neuropsychopharmacology.

[CR70] Ranganath C, Minzenberg MJ, Ragland JD (2008). The Cognitive Neuroscience of Memory Function and Dysfunction in Schizophrenia. Biological Psychiatry.

[CR71] Rasetti R, Weinberger DR (2011). Intermediate phenotypes in psychi- atric disorders. Current Opinion in Genetics & Development.

[CR72] Reininghaus U, Böhnke JR, Hosang G, Farmer A, Burns T, McGuffin P, Bentall RP (2016). Evaluation of the validity and utility of a transdiagnostic psychosis dimension encompassing schizophrenia and bipolar disorder. The British Journal of Psychiatry.

[CR73] Ripke S, Sandler AR, Kendler KS, Levinson DF, Sklar P, Holmans PA (2011). Genome-wide association study identifies five new schizophrenia loci. Nature Genetics.

[CR74] Ripke S, Neale BM, Corvin A, Walter JTR (2014). Schizophrenia Working Group of the Psychiatric Genomics Consortium. Biological insights from 108 schizophrenia-associated genetic loci. Nature.

[CR75] Rucker JJH, McGuffin P (2010). Polygenic heterogeneity: A complex model of genetic inheritance in psychiatric disorders. Biological Psychiatry.

[CR76] Ruderfer DM, Fanous AH, Ripke S, McQuillin A, Amdur RL, Schizophrenia Working Group of Psychiatric Genomics Consortium; Bipolar Disorder Working Group of Psychiatric Genomics Consortium; Cross-Disorder Working Group of Psychiatric Genomics Consortium (2014). Polygenic dissection of diagnosis and clinical dimensions of bipolar disorder and schizophrenia. Molecular Psychiatry.

[CR77] Sklar P, Ripke S, Scott LJ, Andreassen OA, Psychiatric GWAS Consortium Bipolar Disorder Working Group (2011). Large-scale genome-wide association analysis of bipolar disorder identifies a new susceptibility locus near ODZ4. Nature Genetics.

[CR78] Sridharan D, Levitin DJ, Menon V (2008). A critical role for the right fronto-insular cortex in switching between central-executive and default-mode networks. Proceedings of the National Academy of Sciences.

[CR79] Stahl, E., Forstner, A., McQuillin, A., Ripke, S., Bipolar Disorder Working Group of the PGC, Ophoff, R., Scott, L., Cichon, S., Andreassen, O. A., Sklar, P., Kelsoe, J., & Breen, G. (2017). Genome-wide association study identifies 30 loci associated with bipolar disorder. *bioRxiv*, 173062. 10.1101/173062.

[CR80] Stein JL, Medland SE, Vasquez AA (2012). Identification of common variants associated with human hippocampal and intracranial volumes. Nature Genetics.

[CR81] Strakowski SM, Adler CM, Almeida J, Altshuler LL, Blumberg HP, Chang KD (2012). The functional neuroanatomy of bipolar disorder: A consensus model. Bipolar Disorders.

[CR82] Sudlow C, Gallacher J, Allen N, Beral V, Burton P, Danesh J (2015). UK biobank: an open access resource for identifying the causes of a wide range of complex diseases of middle and old age. PLoS Medicine.

[CR83] Sullivan PF (2010). The Psychiatric GWAS Consortium: Big Science Comes to Psychiatry. Neuron.

[CR84] Sullivan, P. F., Agrawal, A., Bulik, C. M., Andreassen, O. A., Børglum, A. D., Breen, G., et al. (2017). Psychiatric genomics: An update and an agenda. *The American Journal of Psychiatry*. 10.1176/appi.ajp.2017.17030283. 10.1176/appi.ajp.2017.17030283 PMC5756100 28969442

[CR85] Sutcliffe G, Harneit A, Tost H, Meyer-Lindenberg A (2016). Neuroimaging Intermediate Phenotypes of Executive Control Dysfunction in Schizophrenia. Biological Psychiatry: Cognitive Neuroscience and Neuroimaging.

[CR86] Tesli M, Kauppi K, Bettella F, Brandt CL, Kaufmann T, Espeseth T (2015). Altered brain activation during emotional face processing in relation to both diagnosis and polygenic risk of bipolar disorder. PLoS One.

[CR87] Townsend J, Altshuler LL (2012). Emotion processing and regulation in bipolar disorder: A review. Bipolar Disorders.

[CR88] Uddin LQ (2014). Salience processing and insular cortical function and dysfunction. Nature Reviews. Neuroscience.

[CR89] Vink M, De Leeuw M, Pouwels R, Van Den Munkhof HE, Kahn RS, Hillegers M (2016). Diminishing striatal activation across adolescent development during reward anticipation in offspring of schizophrenia patients. Schizophrenia Research.

[CR90] Visscher PM, Wray NR, Zhang Q, Sklar P, McCarthy MI, Brown MA, Yang J (2017). 10 Years of GWAS Discovery: Biology, Function, and Translation. American Journal of Human Genetics.

[CR91] Walter H, Schnell K, Erk S, Arnold C, Kirsch P, Esslinger C (2011). Effects of a genome-wide supported psychosis risk variant on neural activation during a theory-of-mind task. Molecular Psychiatry.

[CR92] Walton E, Turner J, Gollub RL, Manoach DS, Yendiki A, Ho BC, Sponheim SR, Calhoun VD, Ehrlich S (2013). Cumulative genetic risk and prefrontal activity in patients with schizophrenia. Schizophrenia Bulletin.

[CR93] Walton E, Geisler D, Lee PH, Hass J, Turner JA, Liu J (2014). Prefrontal inefficiency is associated with polygenic risk for schizophrenia. Schizophrenia Bulletin.

[CR94] Wang T, Zhang X, Li A, Zhu M, Liu S, Qin W (2017). Polygenic risk for five psychiatric disorders and cross-disorder and disorder-specific neural connectivity in two independent populations. NeuroImage Clinical.

[CR95] Weiner MW, Aisen PS, Jack CR, Jagust WJ, Trojanowski JQ, Shaw L, Saykin AJ (2010). The Alzheimer's Disease Neuroimaging Initiative: Progress report and future plans. Alzheimer's & Dementia.

[CR96] Whalley HC, Papmeyer M, Sprooten E, Romaniuk L, Blackwood DH, Glahn DC (2012). The influence of polygenic risk for bipolar disorder on neural activation assessed using fMRI. Translational Psychiatry.

[CR97] Whalley HC, Hall L, Romaniuk L, Macdonald A, Lawrie SM, Sussmann JE (2015). Impact of cross-disorder polygenic risk on frontal brain activation with specific effect of schizophrenia risk. Schizophrenia Research.

[CR98] White TP, Joseph V, Francis ST, Liddle PF (2010). Aberrant salience network (bilateral insula and anterior cingulate cortex) connectivity during information processing in schizophrenia. Schizophrenia Research.

